# Evaluation of multiple transcriptomic gene risk signatures in male breast cancer

**DOI:** 10.1038/s41523-021-00301-0

**Published:** 2021-07-26

**Authors:** Jane Bayani, Coralie Poncet, Cheryl Crozier, Anouk Neven, Tammy Piper, Carrie Cunningham, Monika Sobol, Stefan Aebi, Kim Benstead, Oliver Bogler, Lissandra Dal Lago, Judith Fraser, Florentine Hilbers, Ingrid Hedenfalk, Larissa Korde, Barbro Linderholm, John Martens, Lavinia Middleton, Melissa Murray, Catherine Kelly, Cecilia Nilsson, Monika Nowaczyk, Stephanie Peeters, Aleksandra Peric, Peggy Porter, Carolien Schröder, Isabel T. Rubio, Kathryn J. Ruddy, Christi van Asperen, Danielle Van Den Weyngaert, Carolien van Deurzen, Elise van Leeuwen-Stok, Joanna Vermeij, Eric Winer, Sharon H. Giordano, Fatima Cardoso, John M. S. Bartlett

**Affiliations:** 1 Ontario Institute for Cancer Research, Toronto, ON Canada; 2 Department of Laboratory Medicine and Pathobiology, University of Toronto, Toronto, ON Canada; 3 Department of Statistics, European Organization for Research and Treatment of Cancer (EORTC) Headquarters, Brussels, Belgium; 4 University of Edinburgh, Scotland, UK; 5 Swiss Group for Clinical Cancer Research (SAKK), Bern, Switzerland; 6 Department of Oncology, Cheltenham General Hospital, Cheltenham, UK; 7 Global Academic Programs, University of Texas MD Anderson Cancer Center, Houston, TX USA; 8 Department of Medical Oncology, Jules Bordet Institute, Brussels, Belgium; 9 Beatson West of Scotland Cancer Centre, Glasgow, Scotland UK; 10 Breast International Group, Brussels, Belgium; 11 Division of Oncology, Department of Clinical Sciences, Lund University, Lund, Sweden; 12 University of Washington, Seattle, WA USA; 13 Department of Oncology, Sahlgrenska University Hospital, Gothenburg, Sweden; 14 Medical Oncology, Erasmus Medical Center Rotterdam; BOOG, Rotterdam, The Netherlands; 15 Department Pathology, University of Texas MD Anderson Cancer Center, Houston, TX USA; 16 Department of Pathology, Memorial Sloan Kettering Cancer Center, New York, NY USA; 17 All Ireland Cooperative Oncology Research Group (ICORG), Dublin, Ireland; 18 Department of Oncology, Västmanlands Hospital, Västerås, Sweden; 19 Specialist Hospital. St. Wojciech, Gdansk, Poland; 20 Department of Radiation Oncology Maastro, Masstricht, The Netherlands; 21 Divisions of Human Biology and Public Health Sciences, Fred Hutchinson Cancer Research Center & Department of Pathology, University of Washington, Seattle, WA USA; 22 Department Medical Oncology, University Medical Center Groningen; BOOG, Groningen, The Netherlands; 23 Breast Surgical Unit. Hospital Universitario Vall d´Hebron, Barcelona, Spain; 24 Mayo Clinic, Department of Oncology, Rochester, MN USA; 25 Department of Clinical Genetics, Leiden University Medical Center; BOOG, Leiden, The Netherlands; 26 Department of Radiotherapy, ZNA Middelheim, Antwerpen, Belgium; 27 Department Pathology, Erasmus Medical Center; BOOG, Rotterdam, The Netherlands; 28 Dutch Breast Cancer Research Group (BOOG), Amsterdam, The Netherlands; 29 Department of Medical Oncology, ZNA Jan Palfijn, Merksem, Belgium; 30 Dana-Farber Cancer Institute, Boston, MA USA; 31 University of Texas MD Anderson Cancer Center, Houston, TX USA; 32 Breast Unit, Champalimaud Clinical Center/Champalimaud Foundation; EORTC, Lisbon, Portugal

**Keywords:** Prognostic markers, Tumour biomarkers

## Abstract

Male breast cancer (BCa) is a rare disease accounting for less than 1% of all breast cancers and 1% of all cancers in males. The clinical management is largely extrapolated from female BCa. Several multigene assays are increasingly used to guide clinical treatment decisions in female BCa, however, there are limited data on the utility of these tests in male BCa. Here we present the gene expression results of 381 M0, ER+ve, HER2-ve male BCa patients enrolled in the Part 1 (retrospective analysis) of the International Male Breast Cancer Program. Using a custom NanoString™ panel comprised of the genes from the commercial risk tests Prosigna®, OncotypeDX®, and MammaPrint®, risk scores and intrinsic subtyping data were generated to recapitulate the commercial tests as described by us previously. We also examined the prognostic value of other risk scores such as the Genomic Grade Index (GGI), IHC4-mRNA and our prognostic 95-gene signature. In this sample set of male BCa, we demonstrated prognostic utility on univariate analysis. Across all signatures, patients whose samples were identified as low-risk experienced better outcomes than intermediate-risk, with those classed as high risk experiencing the poorest outcomes. As seen with female BCa, the concordance between tests was poor, with C-index values ranging from 40.3% to 78.2% and Kappa values ranging from 0.17 to 0.58. To our knowledge, this is the largest study of male breast cancers assayed to generate risk scores of the current commercial and academic risk tests demonstrating comparable clinical utility to female BCa.

## Introduction

Male breast cancer (BCa) represents ~1% of all newly diagnosed cancers in men^1^ and ~1% of all breast cancers^2^. Research into this rare disease has been limited, with treatment largely extrapolated from knowledge about female BCa^3^. Surgical management is usually modified radical mastectomy, with a minority of patients being offered breast conserving treatment^4^. Local and systemic treatment is largely informed by treatment indications and regimens used in female breast cancer. However, for adjuvant endocrine treatment, the use of aromatase inhibitors (AIs) alone is not recommended, with tamoxifen for at least 5 years indicated for ER/PgR positive tumors^3,5^. Where AIs are indicated, for example in metastatic male BCa, pituitary blockade with an LHRH agonist or orchiectomy is recommended^6^.

Genetic counselling is recommended for all men with BCa, regardless of family history, due to strong links between male BCa and *BRCA2* mutations, seen in 10% of men with BCa^3,7–9^. Transcriptomic multiparametric assays are now integrated into clinical management guidelines for early female BCa^10^ both as prognostic tools and to identify patients for adjuvant chemotherapy^11,12^. Most guidelines refer exclusively to female BCas with respect to the use of these multiparametric assays. Data relating to these tests in male BC are from retrospective series, most with small numbers of cases limited to evaluation of prognosis for single tests^13–16^; however, we are not aware of any analyses which provide comparative data on multiple signatures with respect to patient outcome. We developed a method to compare signatures using a combined quantitative mRNA array covering key molecular signatures^17^, which have been trained against the results of the same signatures measured by the original methodology^18^. We describe here an analysis of male BCa samples from the EORTC cohort^19^ using these “trained” signatures to compare the result of each test and to determine the association between test result and prognosis in the context of a multi-institutional male BCa cohort.

## Results

### mRNA profiling

Out of the 1483 patients included in the parental study, 699 (47.1%) patients met the eligibility criteria of the research project; the main reasons for exclusion were missing tissue, event status, or event dates (see Supplementary Fig. 1). As previously reported^19^, no evidence for a selection bias due to missing data has been identified. From these, 389 samples had sufficient material for extraction and 381 samples yielded sufficient RNA.

All 381 samples assayed were successfully analyzed using the custom NanoString gene expression panel and passed the quality control (Supplementary Fig. 1, Supplementary Table 1).

### Distribution of gene signatures and concordance

Table 1 details the distribution of risk classification across tests, which markedly differs from one gene signature to another. The proportion of high risk patients ranged from 15.7 to 63.5% and for low risk patients the range was 9.4–53.5% (Table 1). For tests with 3 risk groups, the proportion of intermediate risk cases ranged from 38.3 to 55.6%. Low risk cases ranged from 9.4 to 53.5% of all cases and high risk cases from 15.7 to 63.5%. Cross-tabulations for all test combinations are shown in Supplementary Tables 2–15. When cross-tabulating the pairs of gene signatures, the C-index values ranged from 40.3 to 78.2% and kappa values ranged from 0.17 to 0.58, indicating slight to moderate agreement between the gene signatures (Table 2). Using the Prosigna-trained sub-type classification, 0.8% of cases were Basal-like, 3.4% HER2-enriched, 20.2% Luminal A and 75.6% Luminal B.

**Table 1:** Distribution of risk scores by test.

(*n* = 381)^a^	Oncotype Dx-trained	ROR-PT-trained^b^	IHC4-RNA	MammaPrint-trained	GGI-like risk	Risk-95 gene
Low	129 (33.9)	39 (9.4)	109 (28.6)	204 (53.5)	139 (36.5)	180 (47.2)
Intermediate	146 (38.3)	152 (39.9)	212 (55.6)	–	–	–
High	106 (27.8)	174 (45.7)	60 (15.7)	177 (46.5)	242 (63.5)	(52.8)

*N* = number of cases. Figures in brackets () represent percentages within groups.

^a^19 missing values for Prosigna ROR-PT-trained due to missing tumor size.

^b^Prosigna-trained represents the ROR-PT score, including tumor size.

**Table 2:** Cross-tabulation of risk classification: low or low + intermediate vs high.

	Oncotype-trained	Prosigna-trained	IHC4-RNA	MammaPrint-trained	Risk-95 gene	GGI-like risk
Oncotype Dx-trained						
Prosigna-trained	C = 44.8% (39.6–50.0%) κ_w_ = 0.27 (0.20–0.33) [362]					
IHC4-RNA	C = 66.9% (62.0–71.6%) κ_w_ = 0.58 (0.51–0.64) [381]	C = 40.3% (35.2–45.6%) κ_w_ = 0.19 (0.13–0.25) [362]				
MammaPrint-trained	C = 73.0% (68.2–77.4%) κ_s_ = 0.44 (0.36–0.53) [381]	C = 76.0% (71.2–80.3%) κ_s_ = 0.52 (0.43–0.61) [362]	C = 65.1% (60.1–70.0%) κ_s_ = 0.27 (0.19–0.34) [381]			
Risk-95 gene	C = 67.2% (62.2–71.9%) κ_s_ = 0.36 (0.28–0.44) [381]	C = 74.3% (69.5–78.7%) κ_s_ = 0.49 (0.40–0.58) [362]	C = 58.8% (53.7–63.8%) κ_s_ = 0.21 (0.14–0.27) [381]	C = 75.3% (70.7–79.6%) κ_s_ = 0.51 (0.42–0.59) [381]		
GGI-like risk	C = 62.2% (57.1–67.1%) κ_s_ = 0.33 (0.26–0.39) [381]	C = 78.2% (73.6–82.3%) κ_s_ = 0.57 (0.49–0.65) [362]	C = 50.7% (45.5–55.8%) κ_s_ = 0.17 (0.12–0.22) [381]	C = 75.1% (70.4–79.3%) κ_s_ = 0.51 (0.43–0.59) [381]	C = 75.6% (71.0–79.8%) κ_s_ = 0.50 (0.42–0.59) [381]	

The number between brackets [ ] displays the number of patients with available data. C = Concordance index. κ_s_ = Simple kappa agreement coefficient, estimated for gene signature with 2 risk categories. κ_w_ = Weighted kappa agreement coefficient, estimated for gene signature with 3 risk categories. To assess the concordance and the agreement between one gene signature with 3 risk categories, i.e., low/intermediate/high and one gene signature with 2 risk categories, i.e. low/high, the category “intermediate” risk has been combined with the low risk category. Agreement has been assessed with a simple kappa agreement coefficient. This convention has been applied to compare the following pairs: Oncotype DX-trained vs. MammaPrint-trained, Risk-95 gene, GGI-like risk, ROR-PT-trained -trained vs. MammaPrint-trained, Risk-95 gene, GGI-like risk.

### Survival analysis

Seventy-four patients experienced a locoregional recurrence or distant progression qualifying as events for TTR endpoint, of whom, 55 (74.3%) reported a distant progression as first event. Sixty-one patients experienced a distant recurrence qualifying as events for the TTDR endpoint and 38 patients died after a distant recurrence (BCSS endpoint). Seventy-four patients died in the absence of a distant progression and these deaths were considered as competing risks.

**Fig. 1:**
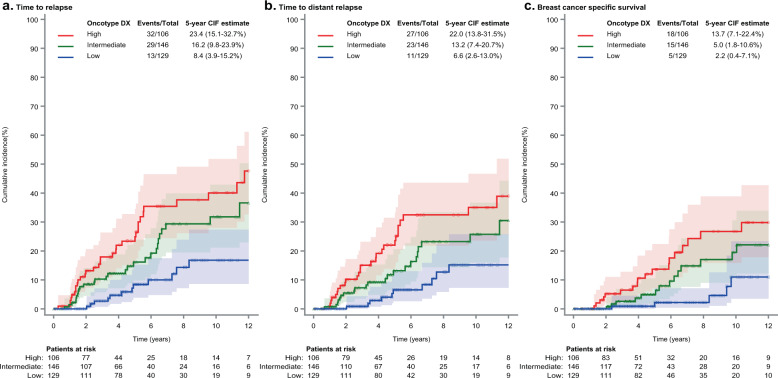
Oncotype DX-trained cumulative incidence of clinical outcomes by risk category. **a** Oncotype DX-trained risk classification for Time to Relapse; **b** Oncotype DX-trained risk classification for Time to Distant Relapse; **c** Oncotype DX-trained risk classification for Breast Cancer Specific Survival. Cumulative incidence rates for low (blue line), intermediate (green line), and high (red line) Oncotype DX-trained results with corresponding 95%CI (shaded areas) estimated by cumulative incidence function accounting for deaths not preceded by a distant relapse as competing risks. Total events/risk group (Events/Total) represent all events observed during follow up (up to 12 years). CIF = 5-year cumulative event frequency (percent) at 5 years with estimated 95% confidence intervals (95% CI).

**Fig. 2:**
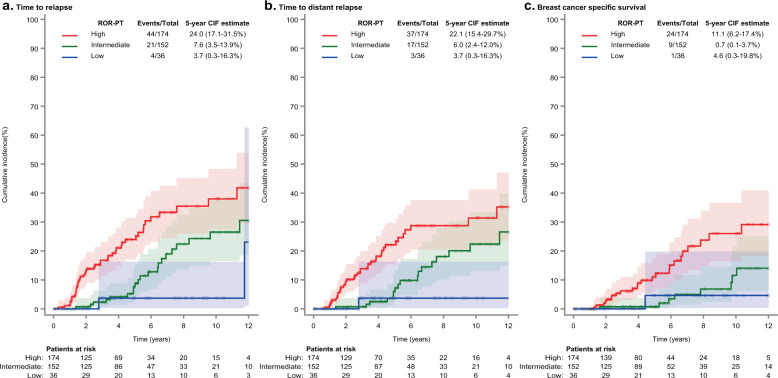
ROR-PT-trained cumulative incidence of clinical outcomes by risk category. **a** ROR-PT-trained risk classification for Time to Relapse; **b** ROR-PT-trained risk classification for Time to Distant Relapse; **c** ROR-PT-trained risk classification for Breast Cancer Specific Survival. Cumulative incidence rates for low (blue line), intermediate (green line), and high (red line) ROR-PT-trained results with corresponding 95%CI (shaded areas) estimated by cumulative incidence function accounting for deaths not preceded by a distant relapse as competing risks. Total events/risk group (Events/Total) represent all events observed during follow up (up to 12 years). CIF = 5-year cumulative event frequency (percent) at 5 years with estimated 95% confidence intervals (95% CI).

Also critical to this analysis, with respect to outcome, when competing risks of deaths not preceded by distant recurrence were accounted for, the cumulative incidence of specific BCa-related events were consistently lower in patients classified as low risk. The 5-year cumulative incidence of locoregional or distant recurrence in low risk patients ranged from 3.7% (95%CI: 0.3–16.3) (Prosigna-trained) to 10.2% (95%CI: 5.6–16.2) (Risk 95-Gene; Figs. 1–2, Supplementary Figs. 2–5) and from 3.7% (95%CI: 0.3–16.3) (Prosigna-trained) to 8.2% (95%CI: 4.1–14.0) (Risk 95-gene) for distant recurrence. For high risk patients, 5-year cumulative incidence of locoregional or distant recurrences ranged from 24.6% (95%CI: 13.8–37.1) (IHC4-RNA) to 20.0% (95%CI: 14.2–26.5) (95-gene) and from 22.1% (95%CI: 15.4–29.7) (Prosigna-trained) to 17.8% (95%CI: 12.2–24.2) (95-gene) for distant recurrence. Regarding BCSS, the 5-year cumulative incidence was below 5% for low risk patients for all tests, whilst for high risk patients the rates ranged between 9.8% (95%CI: 5.8–14.9) and 13.7% (95%CI: 7.1–22.4).

For all signatures evaluated, there was evidence of prognostic information associated with statistically significant risk stratification in low, intermediate (where applicable), and high risk groups (Figs. 1–2, Supplementary Figs. 2–5, Table 3). Each signature provided statistically significant separation of patients into low, intermediate (where appropriate) and high risk groups with respect to TTR, TTDR, and BCSS in univariate analyses (Gray test < 0.01, except for the IHC RNA4 signature) (Figs. 1–2, Supplementary Figs. 2–5). On the contrary, in multivariate-adjusted analyses for TTR and TTDR, the effect of gene-signatures was no longer significant (Table 3).

**Table 3:** 5-year estimate of cumulative incidence and hazard ratio by outcome, risk category and gene signature.

	Risk category	*N*	TTR			TTDR			BCSS	
			5-year cumulative incidence^a^ (95%CI)	Univariate analysis^b^ HR (95%CI)	Multivariate analysis^c^ HR (95%CI)	5-year cumulative incidence^a^ (95%CI)	Univariate analysis^b^ HR (95%CI)	Multivariate analysis^c^ HR (95%CI)	5-year cumulative incidence^a^ (95%CI)	Univariate analysis^b^ HR (95%CI)
Oncotype Dx-trained				*P*-value ≤ 0.001	*P*-value = 0.039		*P*-value = 0.002	*P*-value = 0.051		*P*-value = 0.004
	High	106	23.4% (15.1–32.7)	3.47 (1.84–6.54)	2.32 (0.99–5.45)	22.0% (13.8–31.5)	3.47 (1.74–6.92)	2.29 (0.90–5.80)	13.7% (7.1–22.4)	4.90 (1.83–13.15)
	Intermediate	146	16.2% (9.8–23.9)	2.32 (1.23–4.38)	2.63 (1.25–5.56)	13.2% (7.4–20.7)	2.17 (1.08–4.34)	2.89 (1.23–6.78)	5.0% (1.8–10.6)	3.10 (1.14–8.39)
	Low	129	8.4% (3.9–15.2)	Reference	Reference	6.6% (2.6–13.0)	Reference	Reference	2.0% (0.4–7.1)	Reference
ROR-PT-trained^d^				*P*-value = 0.002	*P*-value = 0.422		*P*-value = 0.004	*P*-value = 0.467		*P*-value = 0.004
	High	174	24.0% (17.1–31.5)	2.89 (1.17–7.16)	2.34 (0.63–8.60)	22.1% (15.4–29.7)	3.15 (1.07–9.26)	2.85 (0.50–16.39)	11.1% (6.2–17.4)	6.01 (0.80–45.36)
	Intermediate	152	7.6% (3.5–13.9)	1.29 (0.50–3.34)	1.84 (0.53–6.38)	6.0% (2.4–12.0)	1.37 (0.45–4.24)	2.24 (0.41–12.35)	0.7% (0.1–3.7)	2.12 (0.27–16.89)
	Low	36	3.7% (0.3–16.3)	Reference	Reference	3.7% (0.3–16.3)	Reference	Reference	4.6% (0.3–19.8) (1 event)	Reference
IHC4 RNA-like				*P*-value = 0.007	*P*-value = 0.085		*P*-value = 0.016	*P*-value = 0.099		*P*-value = 0.056
	High	60	24.6% (13.8–37.1)	3.53 (1.55–8.01)	2.53 (0.91–7.06)	21.4% (11.2–33.9)	3.48 (1.36–8.90)	2.44 (0.73–8.12)	11.9% (4.7–22.8)	4.09 (1.24–13.42)
	Intermediate	212	16.1% (10.9–22.2)	2.58 (1.28–5.22)	2.49 (1.11–5.59)	14.4% (9.4–20.4)	2.85 (1.29–6.27)	2.78 (1.09–7.12)	6.6% (3.4–11.4)	2.82 (0.99–8.03)
	Low	109	8.9% (3.8–16.7)	Reference	Reference	6.6% (2.4–13.8)	Reference	Reference	3.0% (0.5–9.5)	Reference
MammaPrint-trained				*P*-value = 0.002	*P*-value = 0.824		*P*-value = 0.001	*P*-value = 0.594		*P*-value ≤ 0.001
	High	177	21.9% (15.3–29.4)	2.02 (1.29–3.17)	1.09 (0.51–2.36)	20.7% (14.1–28.3)	2.30 (1.40–3.78)	1.25 (0.55–2.83)	12.1% (6.9–18.7)	3.04 (1.59–5.82)
	Low	204	10.0% (5.9–15.4)	Reference	Reference	7.2% (3.8–12.2)	Reference	Reference	2.1% (0.6–5.6)	Reference
95-gene signature				*P*-value ≤ 0.001	*P*-value = 0.149		*P*-value = 0.001	*P*-value = 0.378		*P*-value ≤ 0.001
	High	201	20.0% (14.2–26.5)	2.42 (1.49–3.93)	1.55 (0.86–2.80)	17.8% (12.2–24.2)	2.44 (1.43–4.15)	1.35 (0.69–2.63)	10.9% (6.4–16.7)	3.94 (1.85–8.37)
	Low	180	10.2% (5.6–16.2)	Reference	Reference	8.2% (4.1–14.0)	Reference	Reference	1.7% (0.3–5.5)	Reference
GGI-like				*P*-value =0.001	*P*-value = 0.063		*P*-value = 0.001	*P*-value = 0.059		*P*-value ≤ 0.001
	High	242	20.5% (15.0–26.6)	2.48 (1.43–4.31)	2.03 (0.96–4.27)	18.7% (13.3–24.8)	2.90 (1.55–5.45)	2.32 (0.97–5.55)	9.8% (5.8–14.9)	5.41 (1.95–15.06)
	Low	139	6.7% (2.9–12.6)	Reference	Reference	4.1% (1.3–9.4)	Reference	Reference	0.9% (0.1–4.5)	Reference

*95%CI* 95% confidence interval, *HR* hazard ratio, *N* number of patients, *TTR* time to relapse, *TTDR* time to distant relapse, *BCSS* breast cancer-specific survival, *GGI* Genomic Grade Index.

^a^Cumulative incidence rates and corresponding 95%CI estimated by cumulative incidence function accounting for deaths not preceded by a distant relapse as competing risks.

^b^*P*-value based on Fine and Gray test.

^c^Fine and Gray model adjusted for age, grade, nodal status and tumor size and treatment variables (adjuvant chemotherapy, radiotherapy, endocrine treatment). *P*-value based on Wald test.

^d^ROR-PT-trained uses the trained ROR-PT score, including tumor size; 19 missing values due to missing tumor size.

The 5-year AUC from time-dependent ROC analysis ranged from 0.63 (95%CI: 0.54–0.72) (IHC4-RNA) to 0.72 (95%CI: 0.64–0.80) (Prosigna-trained) for TTR and from 0.65 (95%CI: 0.55–0.74) (IHC4-RNA) to 0.73 (95%CI: 0.64–0.82) (Prosigna-trained) for TTDR. Regarding BCSS, AUC is about 0.75 for each gene signature and is maximal for Risk-95 gene signature (AUC = 0.82, 95%CI: 0.71–0.93) (Supplementary Figs. 6–8).

## Discussion

The use of molecular prognostic assays in female BCa is now well established, but evidence relating to their performance in male BCa patients is sparse. In this study we show, using computational methods to recapitulate multiple BCa prognostic signatures, evidence for the prognostic impact of multiple gene signatures. However, we also show evidence of discordance between signatures, applied to the same case, similar to that seen in female BCa^20^. This study highlights the potential utility of molecular prognostic signatures in male breast cancer but suggests that more research is needed if we are to fully understand the potential value of different approaches to assessing prognosis and directing treatment, using molecular tools, in men with breast cancer.

Critical to our study is the close correlation between the computationally derived “signature trained” scores and true results as shown in our recent paper^18^. For ROR-PT results the correlation coefficient between “trained” and true assay results was 0.93, comparing true to “trained” results showed 90% of cases within the same risk category (low, intermediate, high—see ref. ^18^). Similarly for “Oncotype Dx-trained” results the correlation coefficient between true and “trained” results was 0.87 with 75% of results giving the same risk category (see ref. ^18^) and only 1% of cases disagreeing by more than 1 risk category. For MammaPrint -trained results, which were calculated only as categorical high versus low risk groups, over 90% of cases were classified in the same risk group by “trained” and true results^18^. Full details of these results are reported elsewhere^18^. For Genomic Grade Index and IHC4, we did not have access to actual assay results to enable us to train signatures as we did for other signatures, and for Endopredict we did not have enough genes covered to allow recapitulation of this signature.

Using a common analysis platform and computational methods to recapitulate prognostic scores we found that all molecular signatures tested: Oncotype DX-trained^21,22^, Prosigna-ROR-PT^23,24^, MammaPrint^25–27^, Genomic Grade Index^28^, IHC4-mRNA based IHC4^29^, and our novel 95-gene signature^17^ demonstrated the ability to segregate male BCas into high and low prognostic risk groupings. All signatures were associated with significant differences in 5-year survival for time to recurrence, time to distant relapse and breast cancer-specific survival, between low and high risk groups in univariate analyses (Figs. 1–2, Supplementary Figs. 2–5). However, due to the relatively small number of breast cancer-specific events, we were unable to demonstrate the statistically significant prognostic impact of the majority of signatures in multivariate analysis when adjusting for the following key clinico-pathological covariates:age, grade, nodal status and tumor size and treatment variables (adjuvant chemotherapy, radiotherapy, endocrine treatment). There have been few reports on the utility of prognostic signatures, developed using female BCas, when applied to male BCas and these have largely focused on the utility of Oncotype DX^13,15^. Previous studies showed an 81% 5-year BCa-specific survival for men with recurrence scores >31, slightly lower than the 86.3% 5-year BCSS observed for men with recurrence scores >25 shown in the current study, but commensurate with the different thresholds used^13,15^. In the study by Massarweh et al.^15^, 27.8% of men exhibited RS > 25 compared with 12.4% with scores >31. Given the modest number of events in both studies, we believe our results are broadly comparable to those reported by Massarweh et al.^15^. Results from a similar study by Wang et al.^14^ show a higher all-cause mortality rate in all risk groups, but is limited by failure to exclude competing causes of death, which accounted for almost 50% of events in our current study. This high percentage may be due to the fact that male BC patients are older and have more co-morbidities than their female counterparts. We are unaware of studies reporting patient outcome in male BCa when stratified by tests other than Oncotype DX, making it more challenging to draw comparisons between studies using these molecular assays. With respect to the 50-gene signature driving molecular subtypes (Prosigna/PAM50), a study by Sanchez-Munoz et al.^16^, profiled 67 invasive male BCas using the NanoString panel identifying 60% of cases as Luminal B, 30% Luminal A and 10% HER2-enriched; which is consistent with our findings^18^, however, we are not aware of any studies of male BCas profiled reporting Prosigna risk scores.

As with prior comparisons in female BCa^20^, we demonstrate poor agreement between risk signatures in male BCa with kappa values ranging from 0.17 to 0.58 (Table 2). This modest agreement reinforces observations from larger cohorts of female BCas that different molecular risk scores based on limited mRNA panels may not capture all features related to risk in this population. This conclusion is supported by multiple analyses showing the added value of combining multiple risk signatures in female BCa^30^ and our own recent data highlighting the modest AUCs associated with different molecular signatures^17^ with respect to predicting outcome. Despite different methodologies used, AUCs of time-dependent ROC curves at 5 years for male breast cancer cases fall within the same range of the AUCs reported in female patients^17^. This provides no indication that different cut points for risk would apply to male rather than female breast cancer, however, given the small sample size of the present study it is premature to exclude this possibility entirely. All signatures assessed would appear appropriate for use in male breast cancer patients.

There are several key limitations to our current research project. Firstly, we have used computational methods to calculate the relevant risk signatures rather than the original assays as used in the clinical setting. This limitation is offset in part by the use of a training and validation approach to benchmark results for Oncotype DX, Prosigna, and MammaPrint results against true assay results^18^, but remains a limitation for other tests. Secondly, the analyses were conducted in a retrospective dataset in which not all data were systematically collected in all patients including, the cause of death is not reported for a substantial number of patients leading to a substantial proportion of competing risks. As a result, we have not presented overall survival (all causes) data since this would be confounded by the lack of data as to cause of death in many patients. Despite this study representing one of the largest cohorts of male breast cancers analyzed to date, the sample size and in particular the number of breast cancer related events, limit the statistical power of this analysis. In particular, we were not able to compare the impact of multiple tests performed in sequence due to a lack of statistical power nor were we able to assess the potential impact of tests on chemoprediction. Notwithstanding these limitations we are able to show the ability of a number of existing multiparametric tests (including MammaPrint, Oncotype Dx, Prosigna ROR-PT, Genomic Grade Index and a novel 95-gene signature) to provide useful prognostic information in male breast cancer. These data provide evidence to support the utility of multiple prognostic assays in the context of male breast cancer. Further research to identify the optimal prognostic approach to male breast cancer, perhaps including genomic features such as mutations and copy number alterations, is warranted in addition to investigating the role of intratumoural heterogeneity.

## Methods

### Patients and samples

The retrospective cohort study of the EORTC/TBCRC/BIG/NABCG International Male Breast Cancer Program enrolled male patients with histologically proven BCa, diagnosed between 1990 and 2010, across multiple participating institutions^19^. Ethics approval was provided by the University of Toronto (#30035), a waiver of consent was approved since patient contact was not feasible due to death or loss to follow-up and the research involved no risk to patients whose identify was coded and confidentially protected. Patients with all disease stages (early, locally advanced, and metastatic) were included, irrespective of the treatment received. Availability of a tissue sample (Formalin-Fixed-Paraffin-Embedded—FFPE) of good quality was mandatory for enrollment. Biological material was handled and analyzed centrally according to published guidelines for adoption across BCa clinical trials, conducted by BIG and NABCG, in 2008^31^. Patients in this research project were selected from the retrospective cohort study based on the following exclusion criteria: patients ineligible for the analysis of the parental retrospective cohort, with metastatic (M1/MX) disease, ER-ve per central pathology or local pathology (if central pathology not available), HER2+ve or unknown based on central pathology, insufficient information for assessment of recurrence free survival. In addition, samples with insufficient RNA or which failed the quality control criteria were excluded. All institutions participating in the retrospective cohort study obtained ethical approval from their institutions including consent waivers.

### RNA extraction and expression profiling using NanoString

Profiling of all samples was performed using mRNA extracted and analyzed using the NanoString codeset as described previously^17^ at the Ontario Institute for Cancer Research (OICR).

### Derivation of signature-trained risk stratification scores from candidate assays

Based on our study comparing two different approaches to the generation of simulated risk scores^18^ we selected a training and validation approach based on results obtained from the OPTIMA prelim study^20^ to best fit risk stratification scores generated for this study to those derived from the relevant commercial assay. For all tests we used the suffix “*-trained”* to discriminate the computationally derived assays scores from the commercially derived scores, e.g., Oncotype DX-trained vs. Oncotype-DX®. For each of the commercial test, cases were grouped into pre-defined risk categories according to the cut-points: Oncotype DX—low risk <18, intermediate risk 18–25, high risk ≥25; Prosigna—low risk <40, intermediate risk 40–60, high risk ≥61; MammaPrint—low risk and high risk as described in ref. ^18^. We modified the original cut point for “high risk” for the Oncotype DX test in line with reported results from the TAILORx trial^11,32^ and our previous reported results from OPTIMA prelim^20^. For “Prosigna”, results refer throughout to the ROR-PT risk score in clinical use, which includes tumor pathological size. For the Genomic Grade Index (GGI), the suffix “-like” refers to recapitulation of the risk score as previously described though not trained against a benchmark dataset. The IHC4-mRNA signature is similarly modelled to estimate risk by the transcriptomic expression of ER, PgR, Ki67, and HER2 originally based on the immunohistochemical signature described by Cuzick et al.^33^ The 95-gene signature has been previously described by our group^18^.

### Statistical analyses

Results from the expression profiling using NanoString were provided to EORTC to perform the statistical analysis of clinical data, long term outcomes, and local and central pathology data. Descriptive statistical analysis was performed for patient characteristics, disease characteristics, and treatment(s) administered.

Cross-tabulation of risk classification (low, intermediate—where applicable, high) as defined by the different gene signatures were tabulated to assess concordance and agreement of classification across the different gene signatures. Concordance index and kappa agreement coefficients and their corresponding 95% confidence interval (CI) were estimated. When cross-tabulating gene signatures with different number of categories (i.e., three categories such as low, intermediate, high versus two categories such as high, low), the intermediate category was combined with the low category and Cohen’s simple kappa was estimated while for ternary versus ternary comparisons, the weighted kappa was used.

The prognostic value of the gene signatures was assessed for the following endpoints: time to distant relapse (TTDR) defined as the time until the first distant progression, time to relapse (TTR) defined as the time until the first loco-regional recurrence or distant progression, breast cancer-specific survival (BCSS) defined as the time until breast cancer related death, considering death preceded by a distant relapse. For these endpoints, deaths in the absence of distant relapse are considered as competing risk. The endpoints were calculated from the time of first diagnosis of BCa. Patients without an event for the above endpoints were censored at the last date known alive.

The event rates at 5 years and corresponding 95% confidence intervals were estimated by the cumulative incidence method. Cumulative incidence functions between the risk groups were compared based on the Gray test at a significance level of 0.05. Fine and Gray models were used to estimate the univariate and adjusted hazard ratio (HR) and their corresponding 95%CI. The multivariate models were adjusted for known prognostic clinico-pathological variables (age, grade, nodal status and tumor size) and treatment variables (adjuvant chemotherapy, radiotherapy, endocrine treatment) and the multivariate *p*-value was estimated with the use of a Wald test. Due to the low number of events for BCSS, only univariate analyses were conducted for this endpoint. The proportional hazard assumption was checked graphically using a plot of the log cumulative hazard. The analyses were not adjusted for multiple testing.

The ability of the gene signatures to predict clinical outcome at 5 years was assessed by time-dependent receiver operating characteristic curves (ROC) and the corresponding area under the curve (AUC).The underlying method of ROC curves has been extended to the setting of censored observations and presence of competing risks^34^. Time-dependent ROC curves at 5 years were plotted and the corresponding AUCs estimated for each endpoint (Time to relapse, Time to distant relapse, Breast Cancer-specific survival) and for each gene signature to the exception of MammaPrint. As described previously^18^ when training the algorithm for MammaPrint, only dichotomized risk categories were available preventing any AUC analysis with this signature. Cases were patients that experienced the event of interest in the first five years of follow-up, while controls were defined as patients that were either event-free at 5 years, or experienced a competing event in the first 5 years of follow-up.

Analyses were performed with SAS software, version 9.4 (SAS Institute) and the time-dependent ROC curves were plotted in R, version 4.0.0, with the timeROC package.

### Reporting summary

Further information on research design is available in the Nature Research Reporting Summary linked to this article.

## Supplementary information

Supplementary Information

Reporting Summary

## Data Availability

The data generated and analyzed during this study are described in the following data record: 10.6084/m9.figshare.14616831^35^. The data underlying the Kaplan–Meier survival curves and tables that support the findings of this study are available from European Organisation for the Research and Treatment of Cancer (EORTC). However, the data are not publicly available and restrictions apply to their availability as they were used under license from EORTC for the current study. Data can be made available with the permission of EORTC. Data enquiries can be made to the corresponding author, and data requests can be made at https://www.eortc.org/data-sharing/.

## Code availability

The codes that support these findings are subject to patent applications and restrictions related to licenses. Codes are available from the authors upon reasonable request and with the permission of the Ontario Institute for Cancer Research (OICR).
