# Supplementary material for: Evaluation of multiple transcriptomic gene risk signatures in male breast cancer
Source: NPJ Breast Cancer. 2021 Jul 26;7:98. doi: 10.1038/s41523-021-00301-0 (PMC8313692; doi:10.1038/s41523-021-00301-0)

Supplementary Table 1: Patient characteristics

Patient and Tumor characteristics			
	Analysis population		Total target population (N=931)
	No (N=550)	Yes (N=381)	
	N (%)	N (%)	N (%)
**Age at diagnosis (years)**			
**<= 40**	8 (1.5)	5 (1.3)	13 (1.4)
**41 - 50**	35 (6.4)	31 (8.1)	66 (7.1)
**51 - 65**	179 (32.5)	116 (30.4)	295 (31.7)
**66 - 75**	159 (28.9)	127 (33.3)	286 (30.7)
**> 75**	169 (30.7)	102 (26.8)	271 (29.1)
**Age at diagnosis (years)**			
**Median**	68.6	68.1	68.3
**Range**	25.1 - 98.7	31.6 - 95.8	25.1 - 98.7
**N obs**	550	381	931
**M status***			
**M0**	550 (100.0)	381 (100.0)	931 (100.0)
**LN status (path., clin. if path. is missing)**			
**N0**	331 (60.2)	200 (52.5)	531 (57.0)
**N1**	141 (25.6)	132 (34.6)	273 (29.3)
**N2**	22 (4.0)	26 (6.8)	48 (5.2)
**N3**	12 (2.2)	12 (3.1)	24 (2.6)
**Nx**	44 (8.0)	11 (2.9)	55 (5.9)
**T status**			
**T1**	276 (50.2)	180 (47.2)	456 (49.0)
**T2**	189 (34.4)	160 (42.0)	349 (37.5)
**T3**	8 (1.5)	10 (2.6)	18 (1.9)
**T4**	74 (13.5)	31 (8.1)	105 (11.3)
**Tx**	3 (0.5)	0 (0.0)	3 (0.3)

Supplementary Table 2: Distribution of risk scores by each test.

(n = 381)*	Oncotype Dx-trained	Prosigna-trained**	IHC4-RNA	MammaPrint-trained	GGI-like risk	Risk-95 gene
**Low**	129 (33.9)	39 (9.4)	109 (28.6)	204 (53.5)	139 (36.5)	180 (47.2)
**Intermediate**	146 (38.3)	152 (39.9)	212 (55.6)	-	-	-
**High**	106 (27.8)	174 (45.7)	60 (15.7)	177 (46.5)	242 (63.5)	201 (52.8)

*19 missing values for Prosigna-trained, **Prosigna-trained uses the trained ROR-PT score, including tumour size.

Supplementary Table 3: Cross Tabs :Oncotype Dx-trained and Prosigna-trained

Oncotype Dx-trained vs Prosigna-trained					
	Prosigna_PT-trained				Final sample
N (% of 381)	High	Int.	Low	Missing	
**Oncotype Dx-trained**					
**High**	81 (21.3)	23 (6.0)	2 (0.5)	-	106
**Intermediate**	68 (17.8)	58 (15.2)	11 (2.9)	9 (2.4)	146
**Low**	25 (6.6)	71 (18.6)	23 (6.0)	10 (2.6)	129
**Final sample**	174	152	36	19	381

Supplementary Table 4: Oncotype Dx-trained and IHC4-RNA

Oncotype Dx-trained vs IHC4-RNA				
	IHC4-RNA			Total (N=381)
N (% of 381)	High	Int.	Low	
**Oncotype Dx-trained**				
**High**	52 (13.6)	54 (14.2)	-	106
**Intermediate**	8 (2.1)	116 (30.4)	22 (5.8)	146
**Low**	-	42 (11.0)	87 (22.8)	129
**Final sample**	60	212	109	381

Supplementary Table 5: Prosigna-trained and IHC4-RNA

Prosigna-trained vs IHC4-RNA				
	IHC4-RNA			Final sample
N (% of 381)	High	Int.	Low	
Prosigna_PT-trained				
High	47 (12.3)	105 (27.6)	22 (5.8)	174
Intermediate	11 (2.9)	79 (20.7)	62 (16.3)	152
Low	2 (0.5)	14 (3.7)	20 (5.8)	36
Missing	-	14 (3.7)	5 (1.3)	19
Final sample	60	212	109	381

Supplementary Table 6: Oncotype Dx-trained and MammaPrint-trained

Oncotype Dx-trained vs MammaPrint-trained			
N (% of 381)	MammaPrint-trained		Final sample
	High	Low	
Oncotype Dx-trained			
High	90 (23.6)	16 (4.2)	106
Intermediate	75 (19.7)	71 (18.6)	146
Low	12 (3.1)	117 (30.7)	129
Final sample	177	204	381

Supplementary Table 7: Prosigna-trained and MammaPrint-trained

Prosigna-trained vs MammaPrint-trained			
	MammaPrint-trained		Final sample
N (% of 381)	High	Low	
Prosigna_PT-trained			
High	130 (34.1)	44 (11.5)	174
Intermediate	39 (10.2)	113 (29.7)	152
Low	4 (1.0)	32 (8.4)	36
Missing	4 (1.0)	15 (3.9)	19
Final sample	177	204	381

Supplementary Table 8: IHC4-RNA and MammaPrint-like

IHC4-RNA vs MammaPrint-trained			
	MammaPrint-trained		Final sample
N (% of 381)	High	Low	
**IHC4-RNA**			
High	52 (13.6)	8 (2.1)	60
Intermediate	109 (28.6)	103 (27.0)	212
Low	16 (4.2)	93 (24.4)	109
**Final sample**	177	204	381

Supplementary Table 9: MammaPrint-trained and GG-like risk

MammaPrint-trained vs GGI-like risk			
	GGI risk		Final sample
N (% of 381)	High	Low	
**MammaPrint-trained**			
High	162 (42.5)	15 (3.9)	177
Low	80 (21.0)	124 (32.5)	204
**Final sample**	242	139	381

Supplementary Table 10 MammaPrint-trained and Risk-95 gene

MammaPrint-trained vs Risk-95 gene			
	Risk-95 gene		Final sample
N (% of 381)	High	Low	
**MammaPrint-trained**			
High	142 (37.3)	35 (9.2)	177
Low	59 (15.5)	145 (38.1)	204
**Final sample**	201	180	381

Supplementary Table 11 Oncotype Dx-trained and GGI-like risk

Oncotype Dx-trained vs GGI-like risk			
N (% of 381)	GGI risk		Final sample
	High	Low	
**Oncotype Dx**			
High	102 (26.8)	4 (1.0)	106
Intermediate	109 (28.6)	37 (9.7)	146
Low	31 (8.1)	98 (25.7)	129
**Final sample**	242	139	381

Supplementary Table 12: Prosigna-like and GGI risk

Prosigna-trained vs GGI risk			
	GGI risk		Final sample
N (% of 381)	High	Low	
**Prosigna_PT**			
High	163 (42.8)	11 (2.9)	174
Intermediate	64 (16.8)	88 (23.1)	152
Low	4 (1.0)	32 (8.4)	36
Missing	11 (2.9)	8 (2.1)	19
**Final sample**	242	139	381

Supplementary Table 13 IHC4-RNA and GGI risk

IHC4-RNA vs GGI risk			
	GGI risk		Final sample
N (% of 381)	High	Low	
**IHC4-RNA**			
High	57 (15.0)	3 (0.8)	60
Intermediate	148 (38.8)	64 (16.8)	212
Low	37 (9.7)	72 (18.9)	109
**Final sample**	242	139	381

Supplementary Table 14: Oncotype Dx and Risk-95 gene

Oncotype Dx vs Risk-95 gene			
N (% of 381)	Risk-95 gene		Final sample
	High	Low	
**Oncotype Dx**			
High	91 (23.9)	15 (3.9)	106
Intermediate	81 (21.3)	65 (17.1)	146
Low	29 (7.6)	100 (26.2)	129
**Final sample**	201	180	381

Supplementary Table 15: Prosigna-trained and Risk-95 gene

Prosigna-trained vs Risk-95 gene			
	Risk-95 gene		Final sample
N (% of 381)	High	Low	
**Prosigna_PT**			
High	139 (36.5)	35 (9.2)	174
Intermediate	50 (13.1)	102 (26.8)	152
Low	8 (2.1)	28 (7.3)	36
Missing	4 (1.0)	15 (3.9)	19
**Final sample**	201	180	381

Supplementary Table 16: IHC4-RNA and Risk-95 gene

IHC4-RNA vs Risk-95 gene			
	Risk-95 gene		Final sample
N (% of 381)	High	Low	
**IHC4-RNA**			
High	52 (13.6)	8 (2.1)	60
Intermediate	120 (31.5)	92 (24.1)	212
Low	29 (7.6)	80 (21.0)	109
**Final sample**	201	180	381

Supplementary Figure 1:

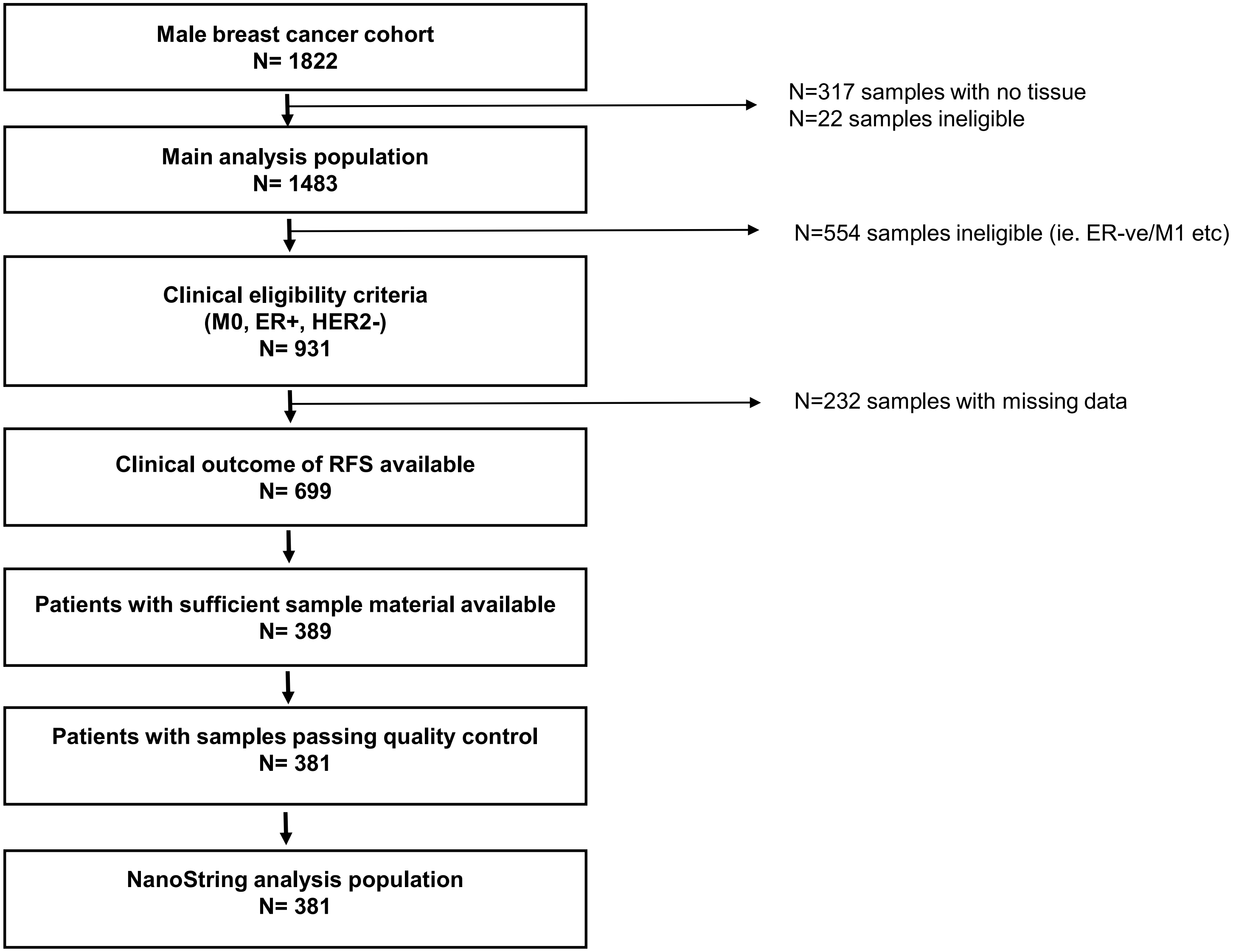

Supplementary Figure 2: IHC4-RNA:

TIME TO RELAPSE

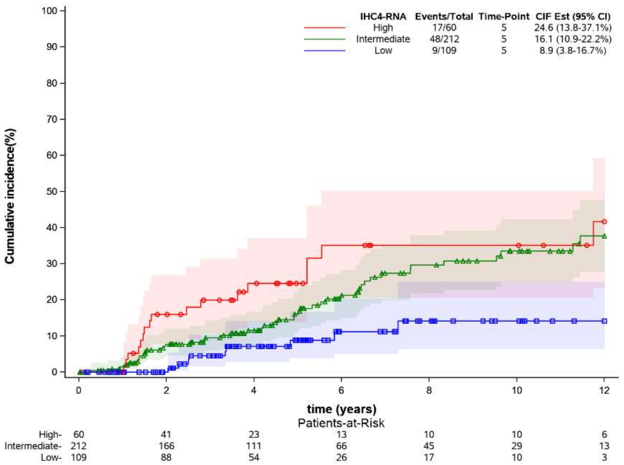

TIME TO DISTANT RELAPSE

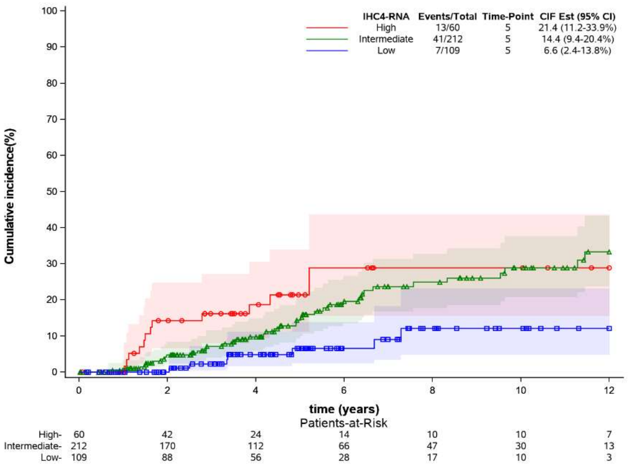

BREAST CANCER SPECIFIC SURVIVAL

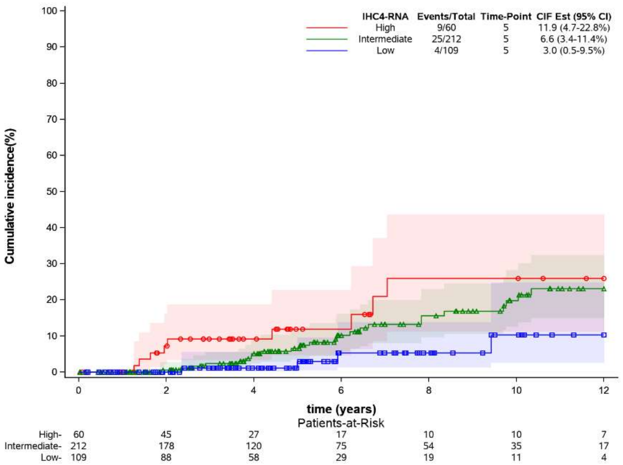

Supplementary Figure 3: MammaPrint-Trained:

TIME TO RELAPSE

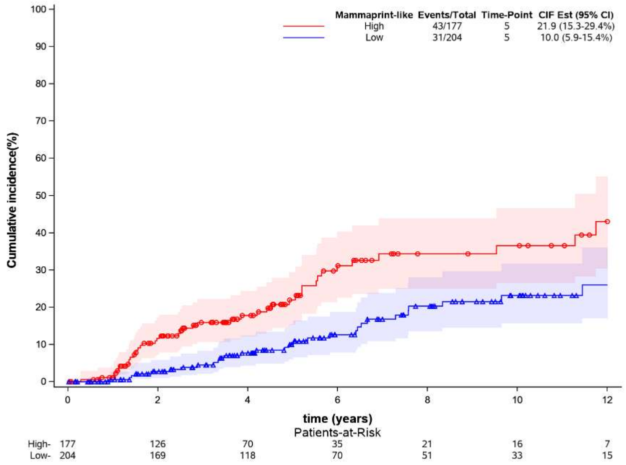

TIME TO DISTANT RELAPSE

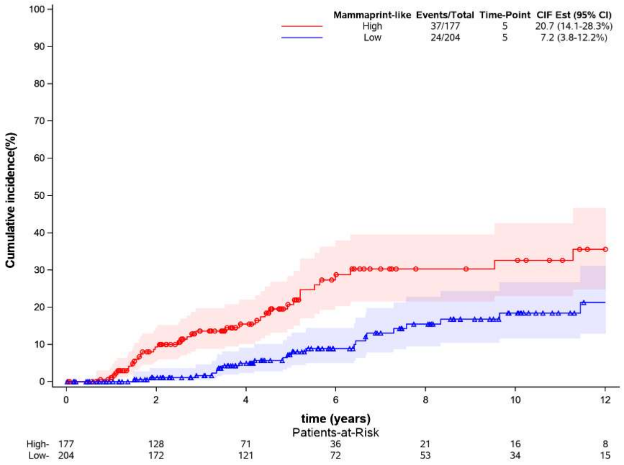

BREAST CANCER SPECIFIC SURVIVAL

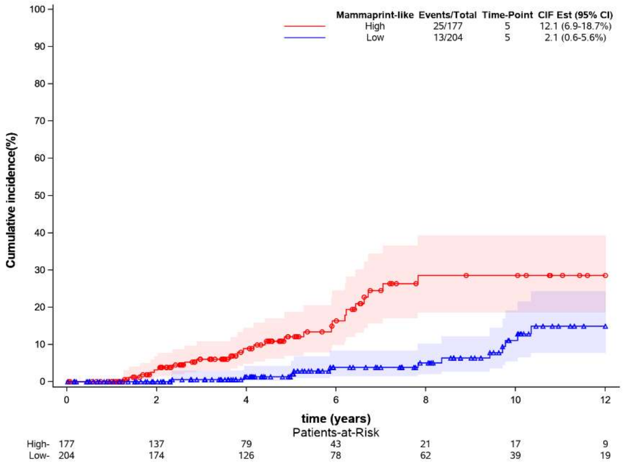

Supplementary Figure 4: 95 Gene-Trained:

TIME TO RELAPSE

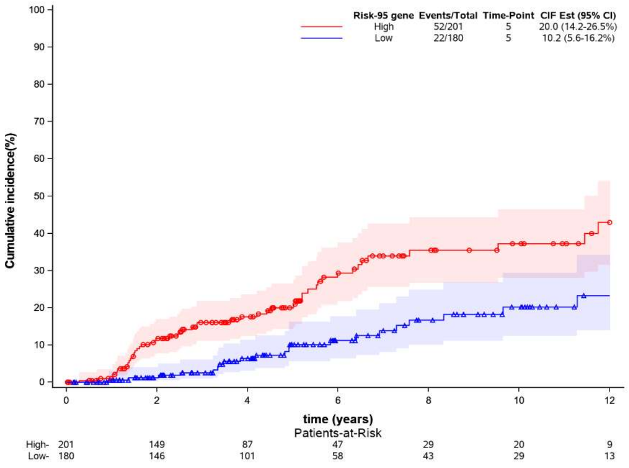

TIME TO DISTANT RELAPSE

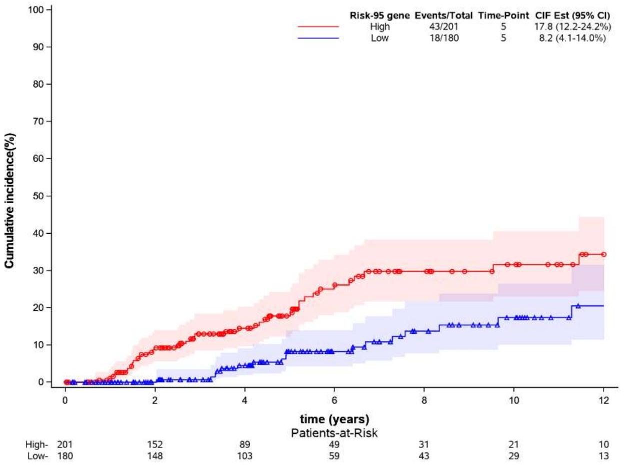

BREAST CANCER SPECIFIC SURVIVAL

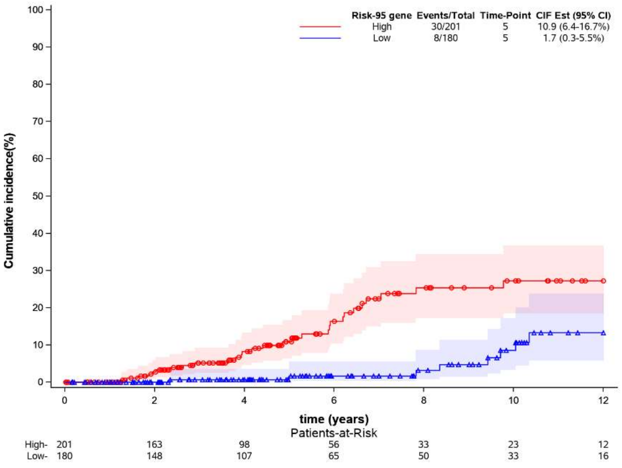

Supplementary Figure 5: GGI-like:

TIME TO RELAPSE

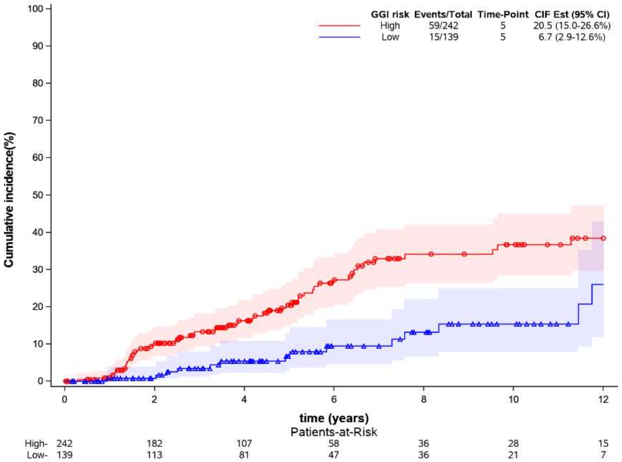

TIME TO DISTANT RELAPSE

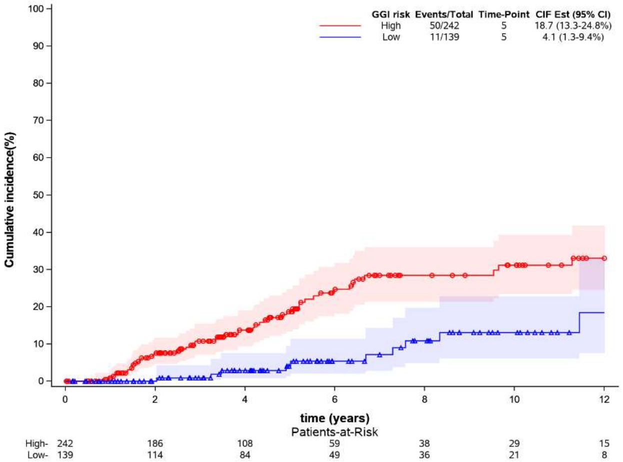

BREAST CANCER SPECIFIC SURVIVAL

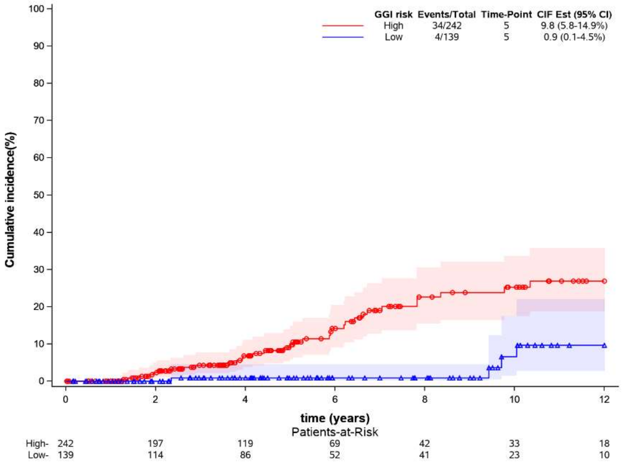

Supplementary Figure 6: Time-dependent ROC curve for time to relapse at 5 years

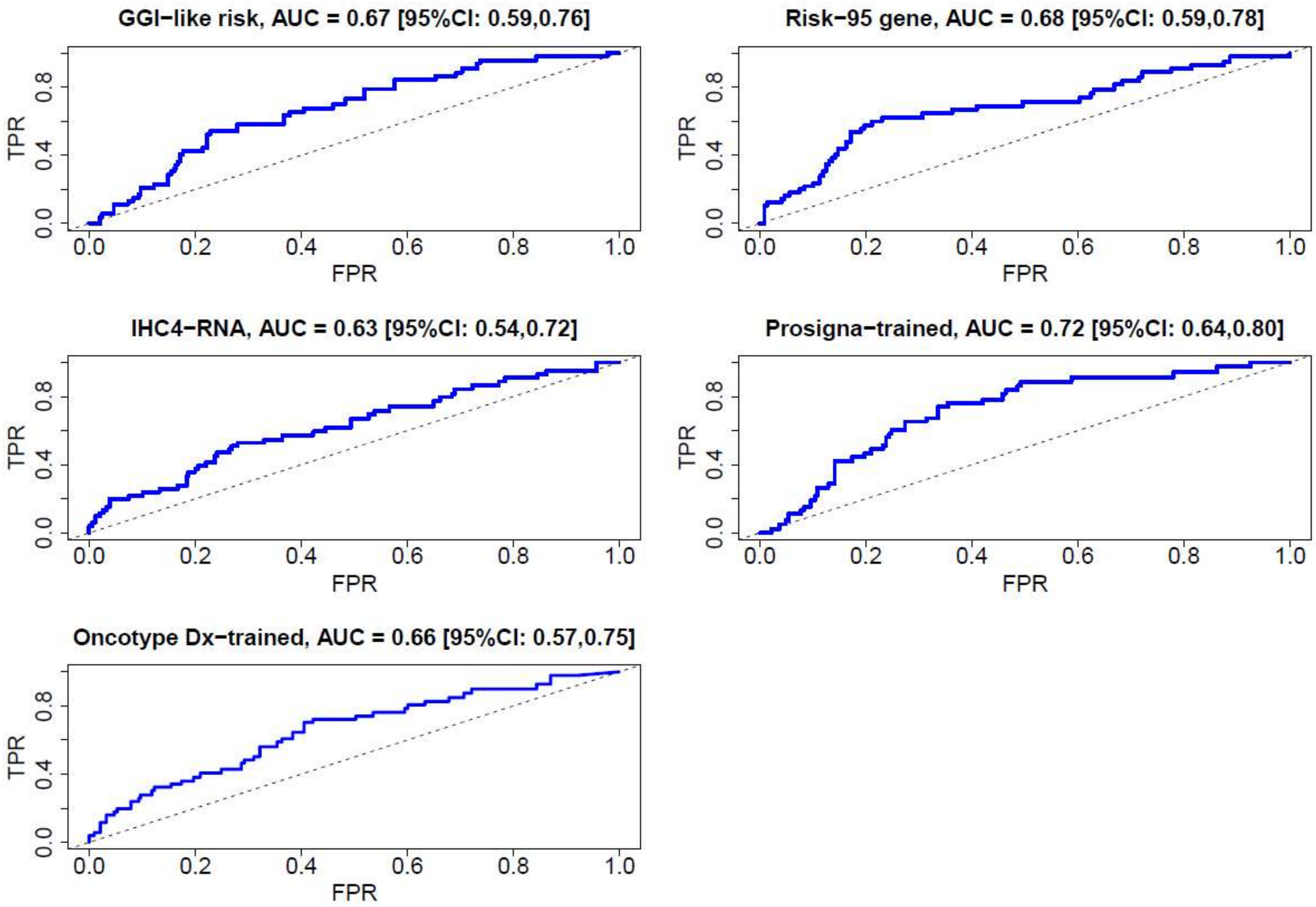

Supplementary Figure 7: Time-dependent ROC curve for time to distant relapse at 5 years

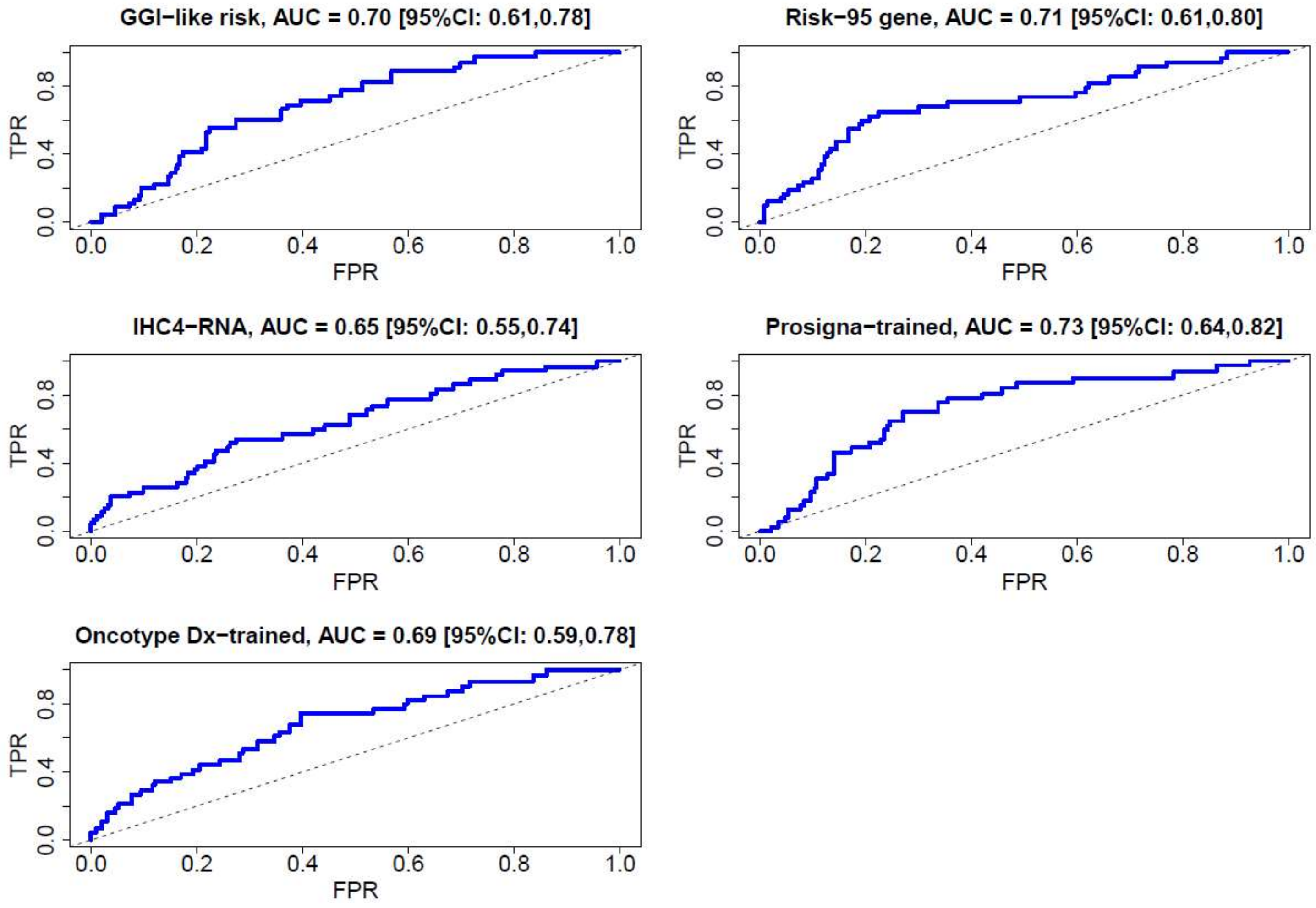

Supplementary Figure 8: Time-dependent ROC curve for breast cancer specific-survival at 5 years

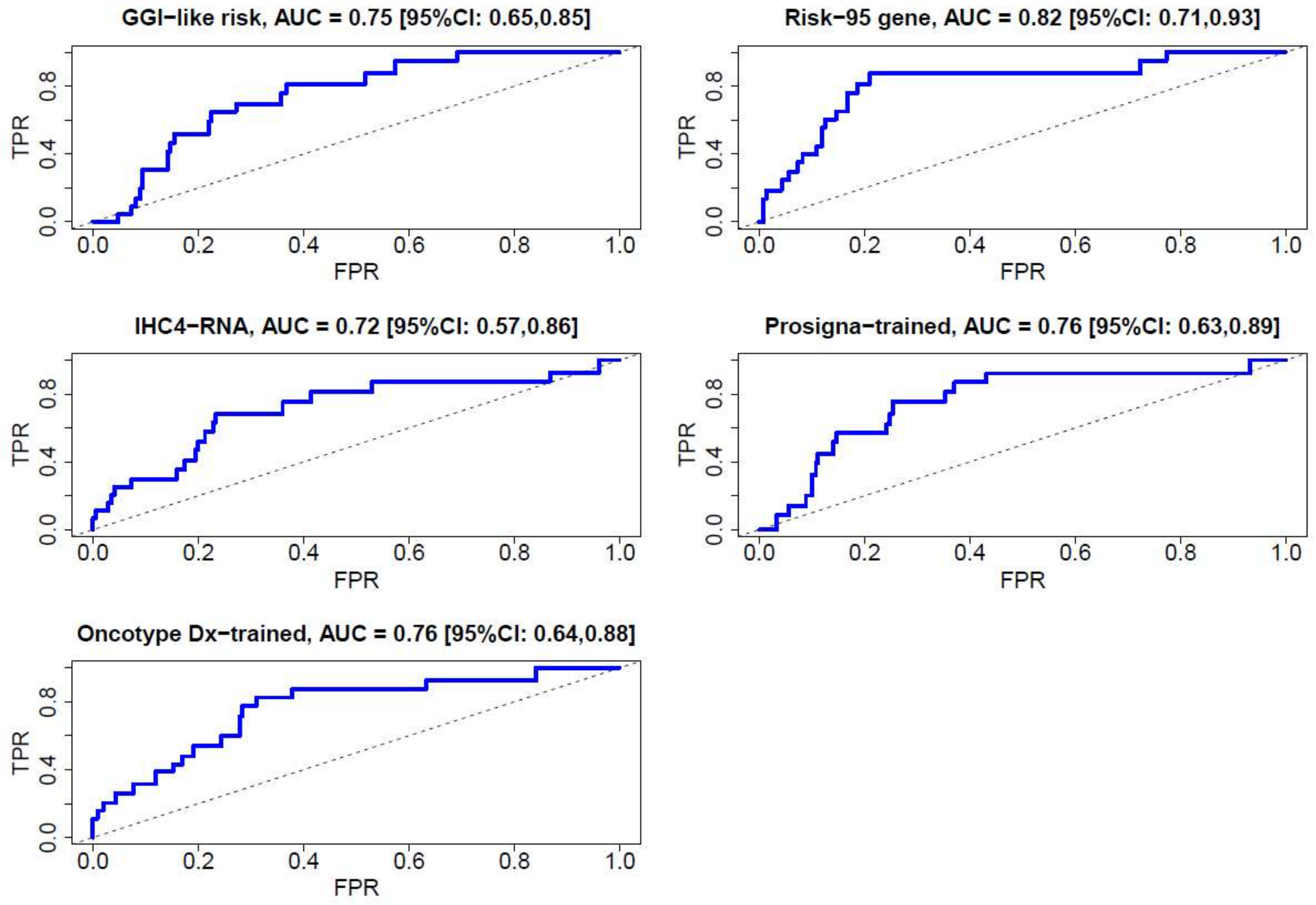

Supplement: Supplementary file 1 — Supplementary Information [file 41523_2021_301_MOESM1_ESM.pdf]
